# Mr. Bacot's Remarks on the Use of Mercury in Sloughing and Phagedenic Chancres

**Published:** 1822-08

**Authors:** 


### Art. III.—Mr. Bacot's *Remarks on the Use of Mercury in Sloughing and Phagedenic Chancres.*

In reading the last Number of the Medico-Chirurgical Review, my attention was particularly attracted by a case of Sloughy Chancre, recorded under the head of "Medical Intelligence," in which it is stated that one-third of the glans penis was destroyed in forty-eight hours, by a "chancerous ulceration assuming a sloughy appearance;" and that, after an unavailing trial of local bleeding, fomentation, &c. no effect being produced upon the disease, "mercury was therefore immediately and rapidly introduced into the system; a sore mouth was produced in thirty-six hours; and that instant the sloughing ulceration ceased, as if arrested by a charm." Such are the facts of this case, to which the following observation is annexed;

"We know that this treatment is not according to orthodox canons; but it has, since the above case, been tried in several others with similar success.

It appears to me that there is much in the above communication that requires to be explained; and I am well convinced that if the practice thus strongly recommended should be adopted in every instance of chancerous ulceration, where the destruction of the parts goes on with great rapidity, without any reference to, or consideration of, the presence of constitutional disturbance, or the precise character of the sore itself, (neither of which are noticed in the above communication,) that great and serious mischief must ensue from such want of discrimination. It must be recollected that I write these observations in total ignorance of the individual case alluded to in the valuable work from which my extracts are taken; but the wide circulation of that work, and the highly important nature of the practical question which is there involved, will be a sufficient apology, I trust, for the following remarks.

Of all the varieties of sore which are to be met with on the genitals, none appear to me to be more clearly marked, and recognized among practitioners by their respective names, than the sloughy and phagedenic chancres, and they are both notoriously under the complete control and influence of mercury. The sloughy chancre, with marked induration about the base and edges, extends itself by a process which may strictly deserve the name of sloughing, and is often excavated in depth very rapidly: whereas, the phagedenic sore spreads quickly indeed, but it spreads rather superficially than in depth; a narrow inflamed edge surrounds it, and it eats its way often round a considerable portion of the penis. In neither of these cases is the pain by any means remarkable; nor does the constitution sympathise with the local complaint. Very different is that more rare form of ulcer, in which the parts are rapidly destroyed. In this case, the destruction appears to be the result of a high degree of inflammation; the pain is exquisite; there is an elevated margin of a deep-red colour round the sore, which is usually situated on the glans penis; and rigors, a furred tongue, and a full and rapid pulse, denote a great degree of constitutional disturbance. The form that this disease assumes, at its first appearance, is that of a small dark-coloured pimple; and an attack of pyrexia commonly precedes the increase of the local complaint. In these cases I have often seen the introduction of mercury attempted, but always with an aggravation of the symptoms; until, by general bleeding, brisk purging, and the use of strict antiphlogistic means, the constitutional symptoms have been removed; in which event, the character of the sore becomes immediately changed, and mercury may afterwards be exhibited for the security of the constitution, if it be thought necessary to do so. It may here be remarked, that, owing to some peculiarity of habit, or other unexplained causes, a simple sore on the penis will sometimes suddenly assume a sloughy appearance, and the health become much deranged. In both these examples, but especially in the former, the exhibition of mercury is found to be practically hurtful, as well as clearly contra-indicated, from a consideration of its stimulating properties.

In the above sketch of what I shall call the inflammatory chancre, I have attempted to describe the most aggravated form of this sore, which I have had occasion to witness several times; but it must be remembered that, although it differs in degree of violence in perhaps every individual case, still the great distinctions as to the irritability of the sore and the derangement of the general health remain the same, and are, I am convinced, of the greatest practical importance.

There is another remark which I would wish to make in this place, which is, that all sores upon the glans penis should be watched with great caution, whether it be thought right to exhibit mercury or not, since ulceration often spreads with great rapidity in that part; and this is especially the case in those instances where the remedy has been pushed too far, or where it begins to excite constitutional erethism, which in some habits it frequently does.

I shall conclude this paper by strongly urging the necessity of a careful examination into the state of health of the patient, in all cases of syphilis, whether primary or secondary. Attention to this particular, in many of the varied and protean forms of eruption especially, will often supersede the necessity of recurring to the use of mercury, and which, if even necessary to be exhibited, would, in all probability, produce more injury than benefit when administered profusely and hastily under such circumstances. Many of those cases of anomalous symptoms that are at present to be met with in a very large, and I am afraid increasing, proportion, being to be ascribed, in my opinion, to the too profuse use of mercury in habits *ill prepared to receive it*, or to its employment under circumstances of exposure to weather or intemperance of living, in which its effects may be truly said to be more fatal than those of the disease for which it is prescribed.

I must not, however, be misunderstood, as wishing to depreciate mercury as a remedy: in syphilis, it is, undoubtedly, our firmest and best reliance; and it is in consequence of that conviction that I am anxious to have its merits properly appreciated, and that it should not have its character degraded by injudicious praise.

*South Audley-street; June 20th*, 1822.

